# Dry Powder Inhaler Formulation of *Lactobacillus rhamnosus GG* Targeting *Pseudomonas aeruginosa* Infection in Bronchiectasis Maintenance Therapy

**DOI:** 10.3390/pharmaceutics16080980

**Published:** 2024-07-25

**Authors:** The-Thien Tran, Wean Sin Cheow, Siyu Pu, Jin-Won Park, Kunn Hadinoto

**Affiliations:** 1 School of Chemistry, Chemical Engineering and Biotechnology, Nanyang Technological University Singapore, Singapore 637459, Singapore; 2 Singapore Institute of Technology, Singapore 138683, Singapore; 3 Department of Chemical and Biomolecular Engineering, Seoul National University of Science and Technology, Seoul 01811, Republic of Korea

**Keywords:** dry powder inhaler, pulmonary drug delivery, lung infection, probiotics

## Abstract

The inhaled delivery of lactic acid bacteria (LAB) probiotics has been demonstrated to exert therapeutic benefits to the lungs due to LAB’s immunomodulatory activities. The development of inhaled probiotics formulation, however, is in its nascent stage limited to nebulized LAB. We developed a dry powder inhaler (DPI) formulation of *lactobacillus rhamnosus GG* (LGG) intended for bronchiectasis maintenance therapy by spray freeze drying (SFD). The optimal DPI formulation (i.e., LGG: mannitol: lactose: leucine = 35: 45: 15: 5 wt.%) was determined based on the aerosolization efficiency (86% emitted dose and 26% respirable fraction) and LGG cell viability post-SFD (7 log CFU/mL per mg powder). The optimal DPI formulation was evaluated and compared to lyophilized naked LGG by its (1) adhesion capacity and cytotoxicity to human lung epithelium cells (i.e., A549 and 16HBE14o- cells) as well as its (2) effectiveness in inhibiting the growth and adhesion of *Pseudomonas aeruginosa* to lung cells. The optimal DPI of LGG exhibited similar non-cytotoxicity and adhesion capacity to lung cells to naked LGG. The DPI of LGG also inhibited the growth and adhesion of *P. aeruginosa* to the lung cells as effectively as the naked LGG. The present work established the feasibility of delivering the LAB probiotic by the DPI platform without adversely affecting LGG’s anti-*pseudomonal* activities.

## 1. Introduction

The oral administration of lactic acid bacteria (LAB) probiotics has been demonstrated in animal studies and in clinical trials to enhance the immunity of the respiratory system, which is attributed to the well-established gut–lung axis effects (i.e., bidirectional communication between the lung and gut systems) [1]. The enhanced immunity has been shown to alleviate the symptoms of various respiratory diseases, such as chronic obstructive pulmonary diseases (COPDs) [2], pneumonia [3], asthma [4], and influenza [5]. During the recent COVID-19 pandemic, the beneficial effects of LAB probiotics were observed in COVID-19 patients, where their restored gut microbiota afforded by probiotics ingestion aided in modulating the lung’s immune response and reducing lung inflammation [6].

In addition to the oral route, the targeted delivery of LAB probiotics to the respiratory tracts has been pursued to further leverage the probiotics’ immunomodulatory activities beyond what are facilitated by the mechanisms of the gut–lung axis [7]. The intranasal administration of LAB probiotics was shown in numerous studies to be effective in preventing and treating upper respiratory tract (URT) infections caused by influenza and syncytial viruses [8,9,10,11]. The effectiveness was attributed to the probiotics’ ability to regulate the productions of macrophages, pro-inflammation cytokines, and early induction of virus antibodies in the URT [12]. Moreover, intranasally delivered LAB probiotics was also shown to prevent allergy-induced asthma [13].

In addition to the intranasal route, LAB probiotics have also been delivered to the lungs by inhalation from the oral cavity with the aim of modulating the lung microbiota [1]. Unlike the intranasal route, however, there are very few studies on inhaled LAB probiotics [14]. The inhaled delivery of LAB probiotics by nebulization was demonstrated in vivo to inhibit lung cancer metastases [15] and reduce inflammation in bronchopulmonary dysplasia [16]. As these two studies [15,16] focused on the impacts of lung microbiota’s modulation, naked LAB probiotics (i.e., without undergoing formulation) were used. Nebulized naked LAB probiotics, however, do not represent a feasible inhaled probiotic product because their aerosolization efficiency and LAB cell viability during storage, which were not characterized in [15,16], are likely to be unsatisfactory.

Recently, Glieca et al. [17] developed a dry powder inhaler (DPI) formulation of LAB probiotics by spray drying with pharmaceutical excipients (i.e., 70: 30 lactose: leucine), which were intended for bronchiectasis maintenance therapy. Individuals suffering from bronchiectasis experience a chronic cycle of inflammation and infection of their respiratory tracts, leading to a progressive lung damage [18]. The recurring infection in bronchiectasis lungs is caused primarily by bacterial pathogen *Pseudomonas aeruginosa* [19]. In Glieca et al. [17], three LAB probiotic strains, i.e., *Lactiplantibacillus plantarum*, *Lacticaseibacillus rhamnosus*, and *Lactobacillus acidophilus*, were investigated. The spray-dried LAB probiotics’ (i) aerosolization efficiency, (ii) cytotoxicity and adhesion toward human lung epithelium cells, and (iii) anti-*pseudomonal* activity were examined in vitro.

The spray-dried LAB powders developed by Glieca et al. [17] exhibited small geometric sizes (≈3–4 μm), which were not much larger than the LAB’s individual size. Nevertheless, they exhibited good aerosolization efficiency without the need for coarse carrier particles. The good aerosolization efficiency could be attributed to leucine—a well-known dispersibility enhancer in DPIs [20]. The inclusion of leucine led to the formation of corrugated surfaces in the spray-dried powders, resulting in reduced interparticle interactions [21]. Nevertheless, the mechanical and thermal stresses imposed on the bacteria arising from the harsh operating condition of spray drying, such as the high drying temperatures (>100 °C), may have detrimental effects on the LAB, especially on thermally labile strains [22].

In the present work, we proposed an alternative method by spray freeze drying (SFD) to prepare a DPI formulation of LAB probiotics intended for bronchiectasis maintenance therapy. The inhaled LAB probiotics was intended to complement the mainstay bronchiectasis therapy by inhaled antibiotics [23]. The *Lactobacillus rhamnosus GG* (LGG) strain, known for its anti-inflammatory [24] and antimicrobial activities in the respiratory tract [25,26], was used as the model LAB probiotics for the DPI formulation. LGG exhibits optimal stability at temperature below 55–65 °C, above which its cellular membrane becomes compromised [27]; hence, low-temperature drying, such as SFD, is preferred for LGG.

In this regard, the SFD method has been widely employed for DPI formulation as it inherently produces large particles (∝10 μm) with a low density, resulting in particles having an ideal aerodynamic diameter for aerosolization and easy particle handling during transport, mixing, and packaging [28]. The large size of SFD particles is attributed to the absence of sprayed droplets’ shrinkage as the frozen aqueous phase undergoes sublimation during the lyophilization stage of SFD [29]. This is unlike spray drying in which sprayed droplets’ shrinkage occurs due to evaporation of the aqueous phase. Moreover, sublimation of the interstitial fluid in the frozen droplets leaves behind pores, resulting in particles having a low density.

To the best of our knowledge, apart from Glieca et al. [17], the present work represented the only other investigation into the DPI formulation of LAB probiotics. Our approach differed from Glieca et al. [17] in terms of the (i) drying method (i.e., SFD versus spray drying), (ii) LAB strains (i.e., LGG versus *Lactiplantibacillus plantarum*, *Lacticaseibacillus rhamnosus*, and *Lactobacillus acidophilus*), and (iii) excipients (i.e., lactose: leucine versus mannitol: lactose: leucine) used.

The first objective of the present work was to determine the optimal excipient composition comprising mannitol, lactose, and leucine that could produce a DPI of LGG exhibiting the optimal (1) in vitro aerosolization efficiency and (2) LGG cell viability after SFD. In this regard, both mannitol and lactose have been commonly used as cryoprotectants in the lyophilization of probiotics [30]. They also represent the regulatory-approved compounds for carrier particles in DPI formulations [31,32]. Furthermore, in bronchiectasis therapy, mannitol is often incorporated into DPI formulations for its mucolytic activity to improve the inhaled drug’s permeability across the thick mucus layer found in bronchiectasis lungs [33]. For this reason, mannitol was used in the present work as the primary excipient with small additions of lactose and leucine to enhance the LGG’s viability and aerosolization efficiency, respectively.

The second objective of the present work was to examine the DPI of LGG with the optimal excipient composition for its (1) in vitro adhesion capacity to human lung epithelium cells (i.e., A549 and 16HBE14o- cells), (2) cytotoxicity toward the lung cells, (3) efficacies in inhibiting *P. aeruginosa* growth and *P. aeruginosa* adhesion to the lung cells, and (4) LGG cell viability and physical stability of the DPI of LGG after prolonged refrigerated storage. The DPI of LGG’s adhesion capacity, cytotoxicity, and anti-*pseudomonal* activity were compared to the lyophilized naked LGG’s (i.e., not formulated with excipients) to examine (if any) the influence of the excipients on the DPI of LGG’s performances.

## 2. Materials and Methods

### 2.1. Materials

*Materials for DPI of LGG preparation and characterizations:* LGG bacteria (ATCC 53103) were purchased from ATCC (Manassas, VA, USA). D-mannitol, α-lactose monohydrate, L-leucine, and phosphate-buffered saline (PBS, pH 7.2) were purchased from Sigma-Aldrich (Singapore, Singapore). Lactobacilli MRS broth and Mueller–Hinton broth (MHB) for bacterial cell culture were purchased from BD Diagnostics (Singapore, Singapore). *Materials for DPI of LGG’s cytotoxicity, lung cell adhesion, and anti-pseudomonal activity tests*: A549 adenocarcinomic human alveolar basal epithelial cells and *P. aeruginosa* PAO1 wild-type strain were purchased from ATCC (Manassas, VA, USA). 16HBE14o- immortalized human bronchial cells (HBE cells in short) were generously gifted by the Lee Kong Chian School of Medicine at the National University of Singapore (Singapore). Penicillin–streptomycin, dimethyl sulfoxide (DMSO), and (3-(4,5-dimethylthiazol-2-yl)-2,5-diphenyltetrazolium bromide) (MTT, 98% purity) were purchased from PAA Laboratories (Pasching, Austria), Alfa Aesar (Ward Hill, MA, USA), and Sigma-Aldrich (Singapore, Singapore), respectively. Dulbecco’s modified Eagle’s medium (DMEM) and fetal bovine serum were purchased from HyClone Laboratories (Logan, UT, USA). Minimum Essential Medium with Eagle’s salt (MEM), L-glutamine (200 mM), and 0.25% trypsin–ethylenediaminetetraacetic acid (EDTA) solution were purchased from Gibco (Grand Island, NY, USA).

### 2.2. Methods

#### 2.2.1. Preparation of DPI of LGG by SFD

LGG bacteria for SFD were cultured in MRS broth for 24 h at ≈10 log colony-forming units (CFUs)/mL, after which the bacteria were recovered by centrifugation at 13,000 RPM for 5 min, which was followed by rinsing with PBS. The rinsed LGG were then lyophilized for 24 h in Alpha 1–2 LD Plus freeze dryer (Martin Christ, Osterode, Germany) at −52 °C and 0.05 mbar to produce dry LGG powders. The feed solution for SFD was prepared by adding the dry LGG powders into aqueous solution of the excipients (i.e., mannitol, lactose, and leucine). Six formulations of DPIs of LGG (i.e., DPI_1 to DPI_6) of varying excipient compositions were investigated at a fixed LGG loading of 35 wt.% (dry mass) (Table 1). A run at a lower LGG loading of 12.5 wt.% (i.e., DPI_7) without lactose was performed to demonstrate the effects of LGG loading and lactose composition on the aerosolization efficiency.

In DPI_1 to DPI_7, the mannitol, lactose, and leucine compositions were varied from 35% to 75%, 5% to 15%, and 5% to 15%, respectively, in an iterative process. First, the effects of the ratio of mannitol to lactose compositions were investigated in DPI_1 to DPI_3 at a fixed leucine composition (i.e., 5 wt.%). Second, after the minimum ratio of mannitol to lactose needed to maintain good LGG cell viability after SFD was determined, the effects of leucine compositions (i.e., 5 to 15 wt.%) were investigated in DPI_3 to DPI_5. Third, the effects of lowering lactose composition and/or LGG loading were examined in DPI_6 to DPI_7.

The SFD of LGG and excipients was performed in four replicates at a fixed total solid feed concentration equal to 5 wt.%. The feed solution was sprayed using a two-fluid atomizer (Büchi B-290, Flawil, Switzerland) into a polypropylene vessel containing 400 mL liquid N_2_. The flow rates of the feed solution and atomizing air were set at 0.25 L/h and 300 L/h, respectively. The distance between the atomizer and the liquid N_2_ surface was set at 10 cm to minimize the loss of the sprayed solution to the container’s walls, following the protocols described in [28]. The resultant frozen particles were then lyophilized at −52 °C and 0.05 mbar for 48 h to produce the DPI of LGG.

#### 2.2.2. LGG Cell Viability after SFD

The number of viable LGG cells after SFD was characterized in four replicates immediately after preparation by dispersing 5 mg DPI of LGG in 20 mL PBS to dissolve the excipients and in turn liberate the bacteria from the excipient matrix. The resultant LGG cell suspension was serially diluted and placed on MRS agar plates for 24 h incubation at 37 °C. Afterwards, the LGG cell viability after SFD was characterized by the reduction in the log CFU/mL of LGG recovered after SFD compared to the initial log CFU/mL supplied in the SFD feed (i.e., 7.9 ± 0.2 log CFU/mL). The percentage LGG cell survival after SFD was calculated by $10^{\left(log{\left(CFU/mL\right)}_{Final}-log{\left(CFU/mL\right)}_{Initial}\right)}\times 100\%$.

#### 2.2.3. Aerosolization Efficiency

The aerosolization efficiency of the DPI of LGG was characterized using a seven-stage Next Generation Impactor (NGI) (Copley Scientific, Nottingham, UK) equipped with an induction port (IP), a pre-separator (PS), and a standardized powder entrainment tube (PET) to simulate an inhaler [34]. The aerosolization efficiency was characterized at inhalation flow rate of 85 L/min to achieve the recommended 4 kPa pressure drop across the PET. The inhalation flow was maintained for 2.8 s to simulate the 4 L volume of air drawn in the typical inhalation of healthy humans. At 85 L/min, the cut-off aerodynamic diameter ($d_{A}$) values for stages 1 to 7 of the NGI were equal to 6.7, 3.7, 2.4, 1.4, 0.8, 0.5, and 0.3 µm, respectively [35]. The mass fraction of the DPI of LGG deposited in each NGI stage was determined from the CFU/mL of LGG recovered in each stage, which was obtained by dispersing the deposited powders in 10 mL of PBS and placing them on MRS agar plates for 24 h incubation at 37 °C.

The aerosolization efficiency was characterized from a minimum of four replicates by the (1) emitted dose (ED), (2) fine particle fraction (FPF), and (3) mass median aerodynamic diameter (MMAD). The ED was defined as the ratio of the mass of inhaled powders successfully emitted off the PET to the initial powder mass in the PET (i.e., 25 mg), whereas FPF was defined as the mass percentage of the emitted powders having $d_{A}$ ≤ 5.0 µm. The FPF was determined by interpolating the cumulative $d_{A}$ distribution curve between stage 1 (cut-off $d_{A}$ = 6.7 µm) and stage 2 (cut-off $d_{A}$ = 3.7 µm) of the NGI. MMAD, defined as the median $d_{A}$ of the emitted powders recovered in stages 1 to 7 of the NGI, was calculated by interpolating the cumulative $d_{A}$ distribution curve at the 50% point.

#### 2.2.4. Physical Characterizations of the Optimal Formulation of DPI of LGG

The morphology of the optimal formulation of DPI of LGG was characterized by scanning electron microscope (SEM) (6390LA, JEOL, Peabody, MA, USA). The geometric size was determined by image analysis using ImageJ version 1.51 software (NIH, Bethesda, MD, USA) with a minimum of 100 particle counts. The bulk density was determined using a tap densitometer at 1000 taps (Quantachrome, Boynton Beach, FL, USA). The solid state of the excipients before and after SFD was examined by powder X-ray diffraction (PXRD) (Bruker D2 Phaser, Bruker, Billerica, MA, USA) operated at 5° ≤ 2θ ≤ 70° with a step size of 0.02°/s. Differential scanning calorimetry (DSC) analysis of the DPI of LGG was performed to examine the solid state of the excipients after SFD using an 822e CryoDSC (Mettler Toledo, Columbus, OH, USA). Fourier transform infrared spectroscopy (FTIR) analysis of the DPI of LGG was performed using a Nicolet™ iS50 FTIR Spectrometer (Thermo Fisher Scientific, Waltam, MA, USA).

#### 2.2.5. Cytotoxicity of DPI of LGG toward A549 and HBE cells

A549 cells were cultivated for 24 h in a 5% CO_2_ incubator at 37 °C in a 24-well tissue culture microplate containing DMEM supplemented with 10% (*w*/*v*) fetal bovine serum and 1% (*w*/*v*) penicillin–streptomycin solutions. Separately, HBE cells were cultivated at 37 °C in a 5% CO_2_ incubator in MEM supplemented with 10% (*w*/*v*) fetal bovine serum, 1% (*w*/*v*) L-glutamine (200 mM), and 1% (*w*/*v*) penicillin–streptomycin solutions. Both A549 and HBE cells were cultivated at a density of 10^5^ cells/well. The DPI of LGG was dispersed in the cell cultivation medium after which it was added to the A549 (or HBE) cells at concentrations between 25 and 500 µg/mL, which translated to LGG densities of 5.5 and 7.0 log CFU/mL, respectively.

The LGG-treated A549 (or HBE) cells were then incubated for 24 h at 37 °C, after which the medium was replaced with fresh serum-free medium containing 100 µL of 5 mg/mL MTT dye solution in PBS. The A549 (or HBE) cells with MTT were then incubated for 4 h at 37 °C. Next, 1 mL DMSO was added to dissolve formazan crystals generated by the viable cells via enzymatic reduction in MTT. The formazan concentration was then determined by the optical density at 550 nm (OD_550_) using an UV-Vis spectrophotometer following the recommended absorbance wavelength provided in the MTT assay kit (i.e., 550–600 nm) and verified with the protocol described in [36]. The A549 (or HBE) cell survivals were determined in four replicates by the ratio of the OD_550_ of the treated cells to the OD_550_ of the control run (i.e., A549/HBE cells not treated with the DPI of LGG). The cytotoxicity tests were also carried out for lyophilized naked LGG at an equivalent CFU/mL to that of the DPI of LGG as well as for the raw excipients at concentrations equal to their compositions in the DPI of LGG (Table 1).

#### 2.2.6. Adhesion of DPI of LGG to A549 and HBE Cells

The adhesion capacities of the DPI of LGG and lyophilized naked LGG to the A549 and HBE cells were characterized in four replicates following the protocols described in [37]. The DPI of LGG and naked LGG were dispersed in the medium used to cultivate the A549 (or HBE) cells at a density of ≈6.0 log CFU/mL. Next, the resultant LGG suspensions were added to the A549 (or HBE) cells and incubated for 3–6 h in a 5% CO_2_ incubator at 37 °C. Afterwards, the A549 (or HBE) cells were rinsed with PBS to remove freely floating LGG, which was followed by adding 0.2 mL of trypsin (0.25% *w*/*v*) for 5 min at 37° C to detach the A549 (or HBE) cells. Next, 0.8 mL PBS was added to the detached cells, and the resultant cell suspension was serially diluted and plated on MRS agar plates, which was followed by incubation at 37 °C for 24 h. Afterwards, the viable LGG cells on the plates were quantified. The LGG adhesion capacities were determined from the ratio of the number of LGG cells recovered from the detached cells to the number of LGG cells initially added to the A549 (or HBE) cells (i.e., 6.0 log CFU/mL).

#### 2.2.7. *P. aeruginosa* Growth Inhibition by DPI of LGG

The ability of the DPI of LGG to inhibit the growth of *P. aeruginosa* PAO1 was evaluated in four replicates by the Kirby–Bauer disk susceptibility tests, as described in [11]. The tests were carried out at two PAO1 and LGG cell density pairings (i.e., 6.0 CFU/mL PAO1 treated with 7.0 log CFU/mL LGG, and 7.0 log CFU/mL PAO1 treated with 8.0 log CFU/mL LGG). Briefly, 100 μL of the PAO1 inoculum was plated on the MRS agar plates, and a Whatman^®^ disc (6 mm diameter) was placed on the agar surface. Next, 5, 10 and 20 μL of the LGG suspension dispersed in deionized water were spotted onto the Whatman^®^ disc. The agar plates were then incubated for 24 h at 37 °C, after which the formed zone of inhibition was characterized by measuring the length between the Whatman^®^ disc’s edge and the edge of the inhibited zone’s halo. The tests were repeated for the lyophilized naked LGG.

#### 2.2.8. Inhibition of *P. aeruginosa* Adhesion to HBE Cells by DPI of LGG

The ability of DPI of LGG to inhibit the adhesion of PAO1 cells to the HBE cells was characterized in four replicates following the protocols described in [26]. Briefly, the DPI of LGG was dispersed in the HBE cell medium at 5.0 or 6.0 log CFU/mL, after which 10 µL of the LGG cell suspension was added to the HBE cells in a 24-well microplate. The plate was then incubated in a 5% CO_2_ incubator at 37 °C for 3 h, during which LGG adhesion took place. Afterwards, 10 µL PAO1 inoculum at 6.0 log CFU/mL was added, and the plate was further incubated for 1 h. Next, the HBE cells were rinsed with PBS to remove freely floating PAO1 and LGG cells. The HBE cells were then detached by adding 0.2 mL of trypsin (0.25% *w*/*v*) for 10 min at 37 °C. Subsequently, 0.8 mL PBS was added to the detached cells, and the resultant cell suspension was serially diluted and plated on MHB agar plates, which was followed by incubation at 37 °C for 24 h. Afterward, the viable PAO1 cells were quantified. The inhibition of PAO1 adhesion was characterized by the ratio of the PAO1 colonies recovered from the detached HBE cells to the value observed in the control run (i.e., HBE cells treated with PAO1 in the absence of LGG). The tests were also performed for the lyophilized naked LGG and blank SFD particles (i.e., only excipient, without LGG).

#### 2.2.9. LGG Cell Viability and DPI Stability after Storage

The optimal formulation of DPI of LGG was stored in a closed container in a refrigerator at 4 °C for three months. The number of viable LGG cells in the DPI after one, two, and three months was characterized in four replicates using the protocols previously described in Section 2.2.2. The LGG cell viability after storage was characterized by the reduction in the log CFU/mL calculated relative to the value at 0 months (i.e., 7.0 ± 0.1 log CFU/mL). In addition to the cell viability, the physical stability of the DPI of LGG during storage was examined by PXRD analysis of the powders after storage.

#### 2.2.10. Statistical Analysis

Statistical significances of the DPI of LGG’s characterization results were determined by a one-way analysis of variance (ANOVA) test (p≤ 0.05) performed in Microsoft Excel version 16.8.

## 3. Results and Discussion

### 3.1. Optimal Excipient Compositions for DPI of LGG

#### 3.1.1. LGG Cell Viability after SFD and Aerosolization Efficiency

In this section, we presented the experimental design to arrive at the optimal formulation of DPI of LGG based on the post-SFD LGG cell survival and aerosolization efficiency. As mentioned before, the LGG loading was fixed at 35 wt.% in all the experimental runs. It should be noted that prior to the iterative approach, a three-factor factorial design approach was attempted to determine the optimal mannitol, lactose, and leucine compositions. However, many of the factorial-design runs led to the production of highly cohesive SFD powders. Thus, the approach was shifted to an iterative process using excipient compositions that did not produce cohesive powders in the factorial design experiments as the starting point.

In the current experimental design, while both mannitol and lactose are commonly used cryoprotectants, a significantly lower mass composition of lactose than mannitol’s was used. The reason was because our preliminary study revealed that SFD-ed lactose existed as amorphous solids, whereas SFD-ed mannitol existed as crystalline solids. Amorphous lactose was notorious for its high hygroscopicity causing low storage stability, difficult handling [38], and in our case, poor aerosolization [39]. Nevertheless, lactose had been widely reported to be an effective cryoprotectant for various LAB, including LGG [40,41,42]. In contrast, mannitol had not been as widely investigated as lactose to function as LAB’s cryoprotectant. Therefore, lactose was included in the SFD formulation at low mass compositions (≤15 wt.%) to strike a balance between aerosolization efficiency and LGG cell viability after SFD. The excipient composition of the first SFD formulation investigated (i.e., DPI_1) comprised 55:5:5 wt.% of mannitol:lactose:leucine.

We first investigated the effects of varying the mannitol-to-lactose ratio at a fixed leucine composition (i.e., 5 wt.%) in DPI_1 to DPI_3. At a mannitol-to-lactose ratio equal to 11 in DPI_1, the LGG cell viability was reduced by −1.7 ± 0.2 log CFU/mL after SFD, which translated to roughly 1.9% cell survival (Table 2). Sample handling-related losses (e.g., losses during transfer) and cell deaths due to spraying and freezing contributed to the cell survival after SFD. Decreasing the mannitol-to-lactose ratio to 5 in DPI_2 and 3 in DPI_3 by increasing the lactose composition to 10 and 15 wt.%, respectively, resulted in smaller reductions in the LGG cell viability after SFD at −1.3 ± 0.2 and −0.9 ± 0.1 log CFU/mL, respectively. This translated to higher LGG cell survivals of approximately 5.5% (DPI_2) and 12.4% (DPI_3), respectively (p < 0.05). The present results hence reaffirmed the good cryoprotective activity of lactose toward LGG.

For the aerosolization efficiency, the DPI of LGG from DPI_1 exhibited mean ED, FPF, and MMAD values of ≈84 ± 2 wt.%, 19 ± 3 wt.%, and 2.2 ± 0.2 μm, respectively (Table 2). The aerosolization efficiencies of DPI_2 and DPI_3 were found to be comparable (p > 0.05) to that of DPI_1 with ED, FPF, MMAD values of 86 ± 3 wt.%, 25 ± 3 wt.%, 1.8 ± 0.3 μm and 86 ± 3 wt.%, 26 ± 4 wt.%, 1.1 ± 0.2 μm, respectively (Table 2). The high ED of DPI_1 to DPI_3 indicated that the DPI of LGG was successfully emitted off the PET with roughly one quarter of the emitted powders possessing $d_{A}$ < 5 μm ideal for deposition in the lower respiratory tract, which is the target site in inhaled bronchiectasis therapy [43]. Among DPI_1 to DPI_3, DPI_3 emerged as the superior formulation owed to its higher LGG cell viability after SFD, where approximately 6.9 log CFU/mL of viable LGG cells was present per mg of the DPI powders.

Compared to the DPI of LAB prepared by spray drying reported in Glieca et al. [17], the FPF of DPI of LGG from DPI_3 (i.e., 26 ± 4%) was about 20% lower, albeit a different inhalation flow rate (i.e., 65 L/min) and inhaler was used in [17]. Therefore, we next investigated the effects of increasing leucine composition aimed at improving the FPF in DPI_3 at a fixed lactose composition of 15 wt.%. The higher leucine compositions of 10 and 15 wt.% in DPI_4 and DPI_5, however, were found to have minimal impacts on ED, FPF, and MMAD (Table 2). The LGG cell viability after SFD became lower as the leucine composition was increased (Table 2). Specifically, the LGG cell viabilities in DPI_4 and DPI_5 were reduced by −1.1 ± 0.1 and −1.4 ± 0.2 log CFU/mL, respectively, after SFD compared to −0.9 ± 0.1 log CFU/mL reduction in DPI_3.

As increasing leucine composition did not lead to any improvement in the aerosolization efficiency, we next attempted lowering the lactose composition from 15 wt.% in DPI_3 to 10 wt.% while conversely increasing the leucine composition to 10 wt.%, which was aimed at making the SFD powders less cohesive (i.e., DPI_6). The results showed that DPI_6 did not exhibit improved aerosolization efficiency compared to DPI_3, while the LGG cell viability after SFD was expectedly lower due to the lower lactose composition used. Hence, among DPI_3 to DPI_6, DPI_3 stood out as the optimal DPI formulation due to its higher LGG cell viability after SFD while exhibiting a similar aerosolization efficiency to the rest.

**Figure A1:**
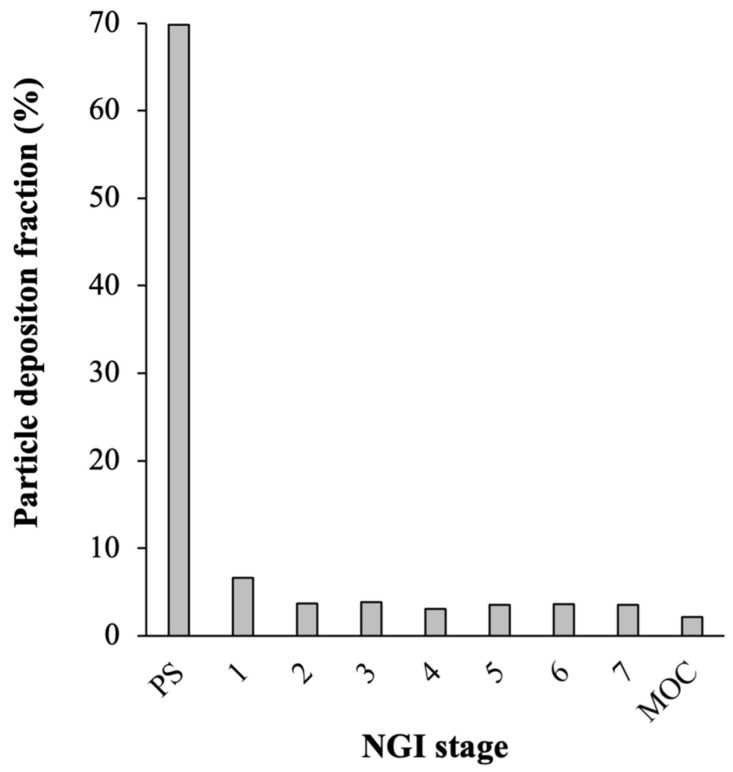
Particle deposition fractions in each stage of the NGI from DPI_3 (PS = pre-separator; MOC = micro-orifice collector). The cut-off aerodynamic diameter ($d_{A}$) for stages 1 to 7 were 6.7, 3.7, 2.4, 1.4, 0.8, 0.5 and 0.3 µm, respectively.

On this note, the SFD powders in DPI_1 to DPI_6 were observed to be cohesive due to the high LGG loading and the inclusion of hygroscopic lactose to maintain LGG cell viability after SFD. The interparticle cohesive forces among the DPI powders prevented them from effectively dispersing mid-air to individual particles upon aerosolization. As a result, a large proportion of the emitted powders deposited as agglomerates in the pre-separator section of the NGI. For illustration, the particle deposition fraction in each stage of the NGI for DPI_3 is presented in Figure A1 in Appendix A.

Therefore, we postulated that the FPF could be increased by lowering the LGG loading and/or lactose composition. In DPI_7, we lowered the LGG loading to 12.5 wt.% and removed lactose from the formulation, resulting in a formulation with higher mannitol and leucine compositions at 75 wt.% and 12.5 wt.%, respectively (Table 1). The results showed that the ED and FPF of DPI_7 were significantly higher at 99 ± 1% and 65 ± 4%, respectively, than that of DPI_3. This signified the greatly superior aerosolization efficiency of DPI_7 attributed to its lower degree of cohesiveness (Table 2).

Nevertheless, the absence of lactose in DPI_7 expectedly resulted in a lower LGG cell viability after SFD with less than 1% cell survival (Table 2). Consequently, when compared to DPI_3, the benefits of having higher ED (≈1.2 × higher) and FPF (≈2.5 × higher) values in DPI_7 were negated by DPI_7’s lower LGG loading (≈2.3 × lower) and lower LGG cell viability after SFD (≈15.5× lower). As a result, at the same dosage, DPI_3 would deliver approximately 12 × more viable LGG cells than DPI_7. Therefore, DPI_3 was selected as the optimal formulation of DPI of LGG to be further characterized for its cytotoxicity, lung cell adhesion, and anti-*pseudomonal* activity.

In this regard, the complete removal of lactose in DPI_7 was performed to test our hypothesis that the hygroscopic lactose was the primary reason for the less-than-ideal FPF despite its importance in ensuring the LGG cell viability after SFD. Prior to DPI_7, we attempted to lower only the LGG loading from 35 wt.% to 25 wt.% while maintaining the same lactose and leucine compositions as DPI_3 (i.e., LGG:mannitol:lactose:leucine = 25:55:15:5 wt.%). The results showed that the SFD powders appeared to be as cohesive as DPI_3; hence, further characterizations were not carried out.

#### 3.1.2. Physical Characteristics of the Optimal Formulation of DPI of LGG

**Figure 1:**
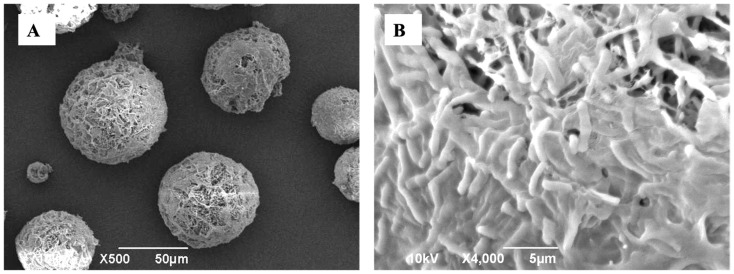
SEM images of the DPI of LGG showing (**A**) its spherical, large, and porous morphology; (**B**) rod-shaped LGG bacteria embedded in the excipient matrix visible on the particle surface.

The DPI of LGG from DPI_3 exhibited a spherical shape with a porous structure characteristic of particles produced by SFD as shown in the SEM image (Figure 1A). A close-up look at the particle surface showed the appearance of rod-shaped LGG bacteria indicating that LGG was not completely enclosed within the excipient matrices, which was not unexpected, as a high LGG loading of 35 wt.% was used (Figure 1B). The mean geometric diameter of the DPI of LGG determined by image analysis was approximately equal to 45 ± 20 μm with a bulk density of 0.07 ± 0.03 g/cm^3^ indicating its porous structure.

**Figure 2:**
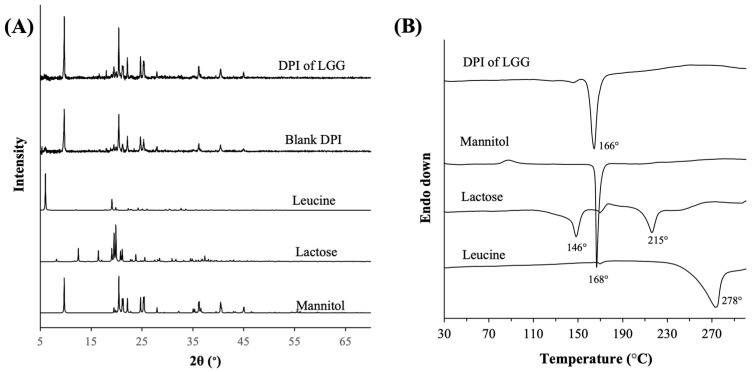
(**A**) PXRD patterns of the DPI of LGG, blank DPI, and the raw excipients; (**B**) DSC thermographs of DPI of LGG and the raw excipients.

PXRD analysis revealed that the DPI of LGG existed as crystalline particles, where the crystallinity was mostly attributed to crystalline mannitol, as evidenced by the peaks in the PXRD spectrum of the DPI of LGG that fully coincided with the strong-intensity peaks of the raw mannitol (Figure 2A). The crystalline form of mannitol in the DPI of LGG boded well for the physical stability of the DPI formulation during its shelf-life, as opposed to amorphous solids which might undergo devitrification, in turn affecting the DPI performances. Importantly, the inclusion of LGG was not found to alter the solid state of the excipients as evidenced by the nearly identical PXRD spectra between the DPI of LGG and the blank DPI (i.e., excipients only).

**Figure A2:**
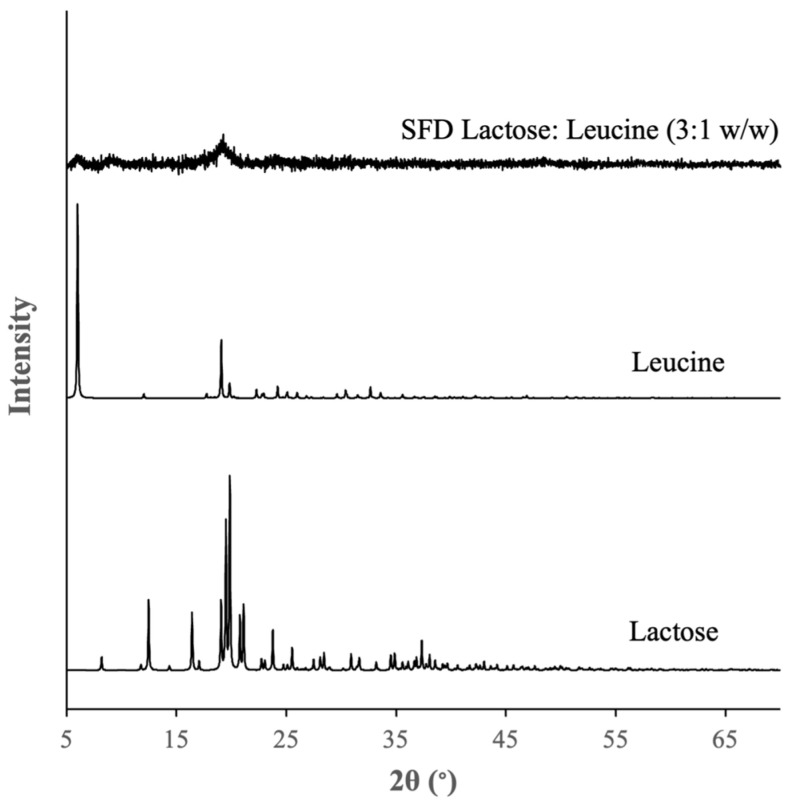
PXRD patterns of spray-freeze-dried lactose and leucine without LGG and mannitol.

The solid state of lactose and leucine could not be clearly discerned from the PXRD patterns due to their much lower compositions compared to that of mannitol. Nevertheless, PXRD analysis of SFD-ed lactose and leucine (3:1 *w*/*w*) without mannitol and LGG did not show the appearance of strong-intensity peaks that typically characterized crystalline solids (Figure A2 in Appendix A). The non-crystalline forms of lactose and leucine in the DPI of LGG were reaffirmed by the DSC analysis (Figure 2B), which showed the absence of endothermic melting point peaks of lactose and leucine that typically appeared at around 215 °C and 278 °C, respectively, in crystalline lactose and leucine. The crystalline form of mannitol was confirmed by the appearance of an endothermic melting point peak at around 166 °C. On this note, the endothermic peak in the DSC thermograph of raw lactose at 146 °C was typically attributed to the loss of crystalline water [44].

**Figure A3:**
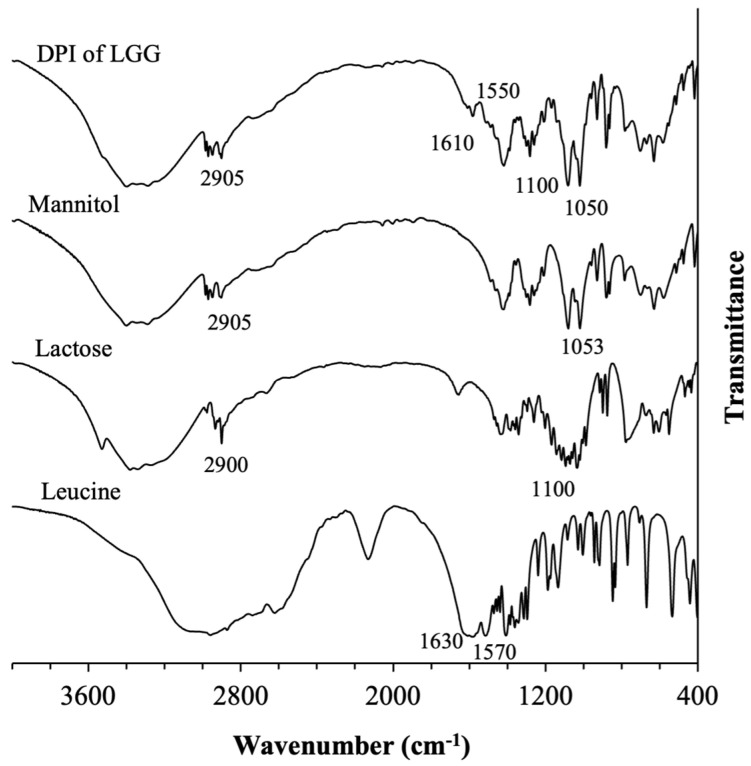
FTIR spectra of the DPI of LGG and the raw excipients.

FTIR analysis of the DPI of LGG expectedly showed the appearances of the characteristic bands of mannitol, lactose, and leucine (Figure A3 in Appendix A). Specifically, the bands at 1050 cm^−1^ and 2905 cm^−1^ were attributed, respectively, to the C-O and C-H stretching vibrations of mannitol molecules in DPI of LGG. The band at 2905 cm^−1^ was also contributed by the C-H stretching vibration of lactose molecules. The multiple small bands appearing between 1100 and 1200 cm^−1^ corresponded to the C-O-C stretching vibrations of the glycosidic bonds in lactose molecules [45]. The bands at 1550 cm^−1^ and 1610 cm^−1^ corresponded, respectively, to the N-H bending and C=O stretching of leucine molecules in the DPI of LGG [46]. The FTIR spectrum of the DPI of LGG also showed a broad band between 3000 and 3500 cm^−1^, which was attributed to the O-H stretching vibrations of the hydroxyl groups present in both mannitol and lactose as well as the N-H stretching vibration of leucine. Similar bands appeared in the FTIR spectra of the raw mannitol, lactose, and leucine.

### 3.2. Performances of the Optimal Formulation of DPI of LGG

#### 3.2.1. Cytotoxicity Toward A549 and HBE Cells

**Figure 3:**
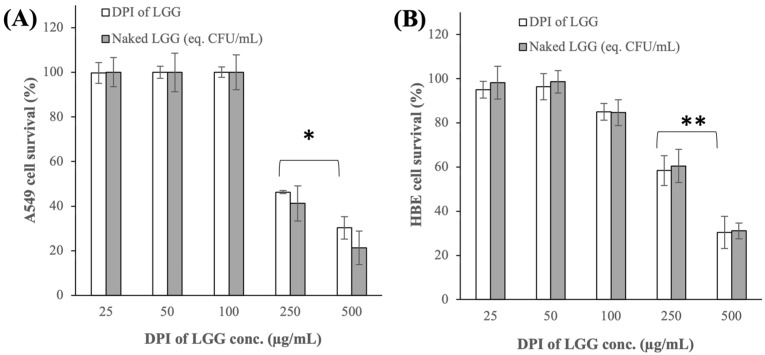
Cytotoxicity of the DPI of LGG and lyophilized naked LGG toward (**A**) A549 cells and (**B**) HBE cells (* p < 0.05, ** p < 0.005).

The DPI of LGG was found to exhibit minimal toxicity toward the A549 cells up to a concentration of 100 μg/mL, which contained approximately 6.0 log CFU/mL of viable LGG cells (Figure 3A). At higher concentrations of 250 and 500 μg/mL, which contained roughly 6.4 and 6.7 log CFU/mL of viable LGG cells, respectively, the A549 cell survival decreased to approximately 46% and 30%, respectively (p < 0.05). To investigate whether the higher cytotoxicity of the DPI of LGG at higher concentrations was caused by the increased excipient concentration, the cytotoxicity of the raw mannitol, lactose, and leucine at concentrations equal to their compositions in the DPI of LGG was examined next.

**Figure A4:**
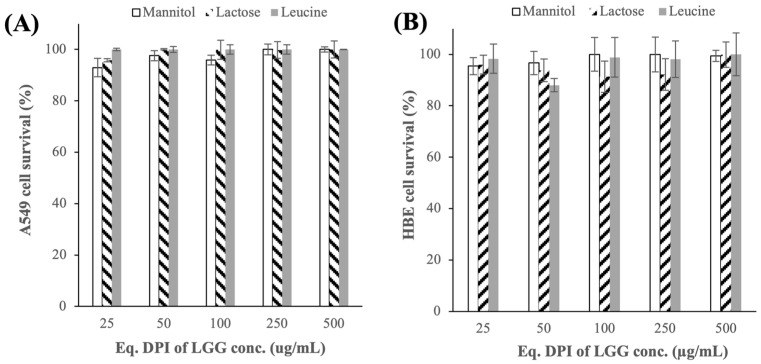
Cytotoxicity of the raw mannitol, lactose, and leucine toward (**A**) A549 cells and (**B**) HBE cells.

The results showed that the raw excipients did not exhibit any cytotoxicity toward the A549 cells even at the highest concentration investigated (Figure A4 in Appendix A). Importantly, the cytotoxicity of the DPI of LGG was found to be highly comparable to that of the lyophilized naked LGG (p > 0.05) (Figure 3A). This finding indicated that the cytotoxicity of DPI of LGG at higher concentrations was caused by excessive LGG colonization of the A549 cells.

A similar trend was observed in the cytotoxicity of the DPI of LGG and the naked LGG toward the HBE cells. Specifically, the HBE cell survivals upon exposures to 250 and 500 μg/mL of the DPI of LGG or the naked LGG decreased to approximately 60% and 30%, respectively (p < 0.005) (Figure 3B). The raw excipients also exhibited minimal cytotoxicity toward the HBE cells (Figure A4 in Appendix A). Based on the cytotoxicity tests’ results, the subsequent characterizations of the DPI of LGG that involved the A549 and HBE cells were carried out at a maximum LGG density of 6.0 log CFU/mL

#### 3.2.2. Adhesion Capacity to A549 and HBE Cells

**Figure 4:**
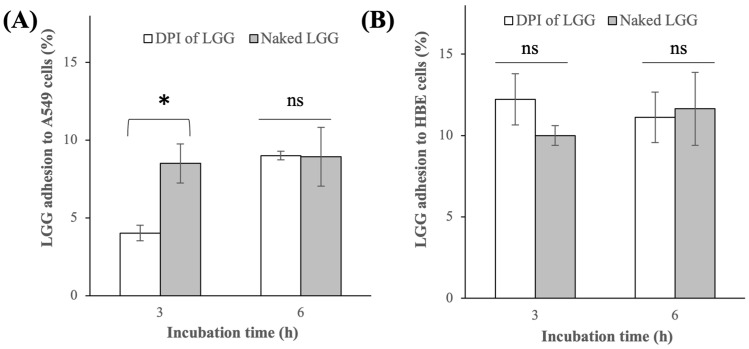
Adhesion capacity of the DPI of LGG and lyophilized naked LGG to (**A**) A549 cells and (**B**) HBE cells (* p < 0.05, ns = not statistically significant).

The ability of LGG cells to adhere to the lung cells below their cytotoxicity threshold was characterized as it signified LGG’s prolonged presence in the lungs, which in turn would bring therapeutic benefits to the lungs. For bacterial microbes, they were typically cleared from the lower respiratory tract by mucociliary clearance within 6 h [47]; thereby, the lung cell adhesion was studied in the present work after 6 h of incubation time. The results showed that the DPI of LGG exhibited comparable adhesion capacities to the A549 cells, as the lyophilized naked LGG with 9 ± 0.3% of the LGG cells added ended up adhered to the A549 cells after 6 h (p > 0.05) (Figure 4A). It was worth mentioning that the adhesion capacity of the naked LGG reported in the present work was comparable to the previous result of [48], which reported the adhesion capacity of two different l. rhamnosus strains (naked forms) to A549 cells to be in the range of 2–3% after 2 h.

Similar trends were observed in the adhesion capacities of the DPI of LGG and naked LGG to the HBE cells. The DPI of LGG and naked LGG exhibited comparable adhesion capacities with 11 ± 1.5% of LGG being adhered to the HBE cells after 6 h (p > 0.05) (Figure 4B). The similarity in the adhesion results between the DPI of LGG and the naked LGG signified the minimal influence of the excipients had on the LGG adhesion to the lung cells. In this regard, at the ≈10% adhesion capacity reported here, approximately 5 log CFU/mL of viable LGG cells adhered to the A549 and HBE cells after 6 h.

A closer look at the adhesion capacity results before 6 h revealed that the adhesion of the DPI of LGG to the A549 cells took place at a slower rate than the naked LGG. Specifically, only 4 ± 0.5% of the bacteria from the DPI of LGG adhered to the A549 cells after 3 h compared to roughly 9% for the naked LGG after the same period. However, the slower adhesion rate of the DPI of LGG after 3 h was not observed with the HBE cells. The difference in the adhesion rates between the two types of lung cells suggested that the DPI of LGG interacted differently with the A549 cells compared to with the HBE cells. The adhesion rate of the naked LGG, on the other hand, did not exhibit a significant difference between the A549 and HBE cells, suggesting that the naked LGG, unlike the DPI of LGG, interacted in a similar manner with the two lung cells. The factors that influenced the adhesion rate of the DPI of LGG to lung cells merit an investigation in the future.

#### 3.2.3. Growth Inhibition of *P. aeruginosa* PAO1

**Figure 5:**
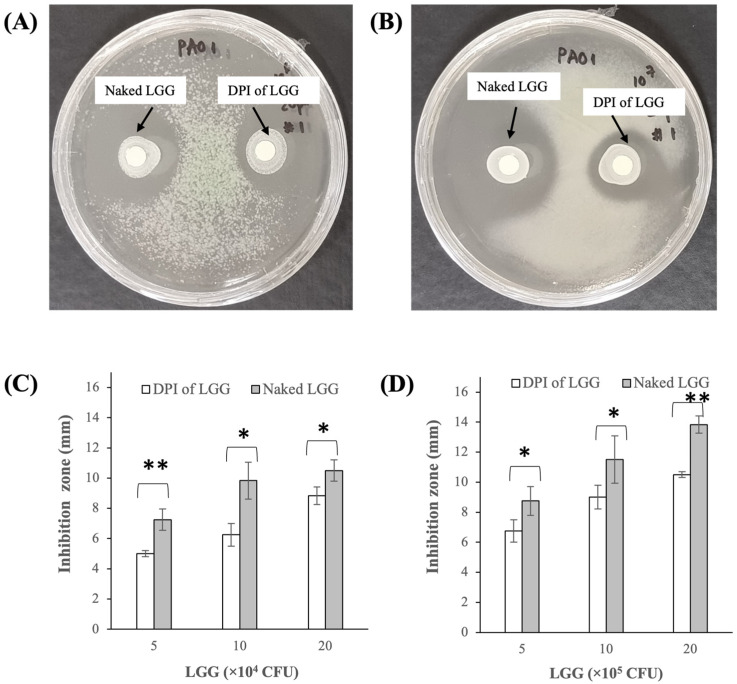
Disk susceptibility test results showing growth inhibition of *P. aeruginosa* PAO1 cultivated at (**A**) 6.0 and (**B**) 7.0 log CFU/mL inoculums upon PAO1 exposures to the DPI of LGG and lyophilized naked LGG at different concentrations; length of the inhibition zones for the (**C**) 6.0 and (**D**) 7.0 log CFU/mL PAO1 inoculums (* p < 0.05, ** p < 0.005).

The DPI of LGG was able to inhibit the *P. aeruginosa* PAO1 growth as evidenced by the appearance of circular inhibition zones in the disk susceptibility tests performed at 6.0 and 7.0 log CFU/mL PAO1 inoculums shown in Figure 5A and Figure 5B, respectively. The sizes of the inhibition zones expectedly increased as the LGG exposure was increased from 5 to 20 ${\times 10}^{4}$ CFU for the 6.0 log CFU/mL PAO1 inoculum (Figure 5C) and from 5 to 20 ${\times 10}^{5}$ CFU for the 7.0 log CFU/mL PAO1 inoculum (Figure 5D).

Compared to the lyophilized naked LGG, the DPI of LGG produced smaller inhibition zones at about 15–35% lower depending on the LGG exposures, indicating the lower bacteriostatic activity of the DPI of LGG for the two PAO1 inoculum values investigated. The DPI of LGG’s lower bacteriostatic activity could be attributed to the presence of excipients, particularly mannitol and lactose, that might alter the growth rates of both PAO1 and LGG during the 24 h incubation period. Importantly, the growth inhibition activity of LGG reported here was consistent with several studies that had reported bacteriostatic activities of other lactobacillus strains against *P. aeruginosa* [49,50].

#### 3.2.4. Inhibition of *P. aeruginosa* PAO1 Adhesion to HBE Cells

**Figure 6:**
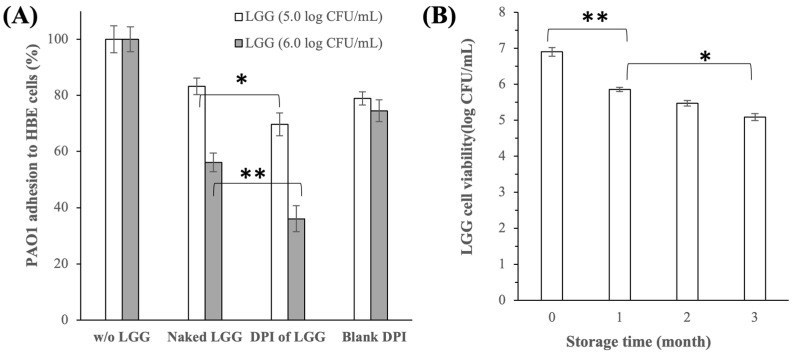
(**A**) Inhibition of the *P. aeruginosa* PAO1 adhesion to HBE cells in the presence of the DPI of LGG, blank DPI, and lyophilized naked LGG; (**B**) LGG cell viability after 0, 1, 2, and 3 months storage at 4 °C (* p < 0.05, ** p < 0.005).

In addition to growth inhibition, the DPI of LGG was also demonstrated to inhibit the adhesion of PAO1 bacteria to the HBE cells. Relative to the control run (i.e., without LGG), the PAO1 adhesions were reduced by roughly 30% and 64% in the presence of DPI of LGG at 5.0 and 6.0 log CFU/mL, respectively (p < 0.05) (Figure 6A). For comparison, PAO1 adhesions in the presence of lyophilized naked LGG at 5.0 and 6.0 log CFU/mL were reduced by roughly 17% and 44%, respectively (p < 0.05) (Figure 6A). Compared to the naked LGG, the greater inhibition of PAO1 adhesion by the DPI of LGG (p < 0.05) was attributed to the contributions from the excipients as demonstrated by the results of the blank DPI (i.e., excipient only, without LGG). Specifically, in the presence of the blank DPI at equivalent concentrations to the excipient concentrations in the DPI of LGG at 5.0 and 6.0 log CFU/mL, the PAO1 adhesions were reduced by approximately 20–25% (Figure 6A). As the presence of both LGG and excipients had inhibitory effects on the PAO1 adhesion to the HBE cells, albeit at varying degrees, their simultaneous presence in the DPI of LGG resulted in the higher PAO1 adhesion inhibition compared to the naked LGG.

#### 3.2.5. LGG Cell Viability and DPI of LGG Stability after Storage

**Figure A5:**
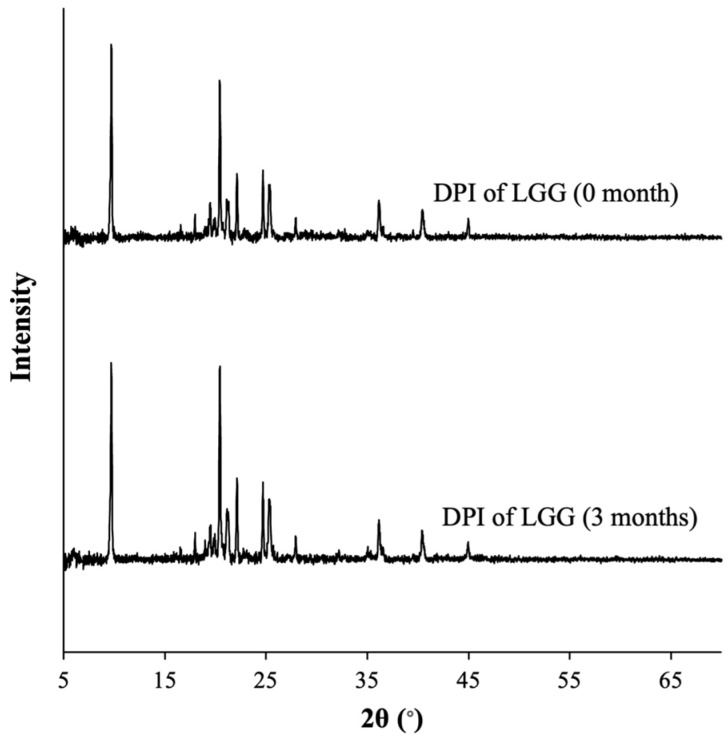
PXRD analysis of the DPI of LGG before and after storage.

The LGG cell viability in the DPI of LGG decreased by approximately −1.0, −1.4, and −1.8 log CFU/mL after storage of one, two, and three months at 4 °C, respectively (Figure 6B). This translated to roughly 5.1 log CFU/mL of viable LGG cells per mg of DPI dosage after 3 months of storage, which would be more than sufficient for the DPI of LGG to inhibit the growth and lung cell adhesion of *P. aeruginosa* as demonstrated in the previous sections. With regard to the solid-state stability of the excipients, PXRD analysis of the DPI of LGG after three months of storage at 4 °C showed that the DPI of LGG maintained its solid forms during storage without signs of devitrification of the non-crystalline lactose and leucine (Figure A5 in Appendix A).

## 4. Conclusions

The present work developed a DPI formulation of LGG intended for bronchiectasis maintenance therapy by SFD using mannitol, lactose, and leucine as the excipients. The optimal DPI formulation contained 35 wt.% LGG loading, which translated to approximately 7 log CFU/mL of viable LGG per mg of the SFD powders before it was reduced to roughly 5 log CFU/mL after three months storage at 4 °C. The optimal formulation of DPI of LGG exhibited ED and FPF values of ≈86 ± 2% and 26 ± 4%, respectively, with an MMAD of ≈1.1 ± 0.2 μm. The DPI of LGG was found to exhibit similar non-cytotoxicity to the A549 and HBE cells to the naked LGG at exposures of up to 6 log CFU/mL. The DPI of LGG also exhibited similar adhesion capacities to the A549 and HBE cells to the naked LGG, signifying the minimal effects of the excipients on the DPI of LGG’s interactions with the lung cells. The growth of *P. aeruginosa* and its adhesion to the HBE cells were inhibited in the presence of the DPI of LGG. Compared to the naked LGG, the DPI of LGG exhibited superior inhibition of *P. aeruginosa* adhesion to the HBE cells, as the excipients themselves also exhibited inhibition activity of *P. aeruginosa* adhesion. In conclusion, the present work successfully demonstrated the feasibility of delivering LAB probiotics to the lower respiratory tract using the DPI platform via SFD without adversely affecting the probiotics’ interactions with the lung cells and effectiveness against *P. aeruginosa.* Nevertheless, further optimizations covering not only the excipient composition, but also the LGG loading, need to be performed in the future to improve the FPF of DPI of LGG without compromising the LGG cell viability.

## Figures and Tables

**Table 1:** DPI of LGG formulations investigated.

Formulation	LGG (Dry Mass) (wt.%)	Mannitol (wt.%)	Lactose (wt.%)	Leucine (wt.%)
DPI_1	35	55	5	5
DPI_2	35	50	10	5
DPI_3	35	45	15	5
DPI_4	35	40	15	10
DPI_5	35	35	15	15
DPI_6	35	45	10	10
DPI_7	12.5	75	0	12.5

**Table 2:** LGG cell viability after SFD and aerosolization efficiency.

Formulation	Cell Viability Reduction (log CFU/mL)	LGG Survival Rate (%)	ED (wt.%)	FPF (wt.%)	MMAD (μm)
DPI_1	−1.7 ± 0.2	1.9	84 ± 2	19 ± 3	2.2 ± 0.2
DPI_2	−1.3 ± 0.2	5.5	86 ± 3	25 ± 3	1.8 ± 0.3
DPI_3	−0.9 ± 0.1	12.4	86 ± 2	26 ± 4	1.1 ± 0.2
DPI_4	−1.1 ± 0.1	7.8	86 ± 1	28 ± 3	0.8 ± 0.4
DPI_5	−1.4 ± 0.2	3.8	83 ± 3	27 ± 2	0.9 ± 0.3
DPI_6	−1.3 ± 0.2	5.5	87 ± 2	28 ± 2	0.6 ± 0.1
DPI_7	−2.1 ± 0.1	0.8	99 ± 1	65 ± 4	2.4 ± 0.8

## Data Availability

The raw data supporting the conclusions of this article will be made available by the authors on request.
